# Preliminary Safety and Potential Effect of 6B11-OCIK Adoptive Cell Therapy Against Platinum-Resistant Recurrent or Refractory Ovarian Cancer

**DOI:** 10.3389/fimmu.2021.707468

**Published:** 2021-08-02

**Authors:** Hongyan Cheng, Ruiqiong Ma, Shang Wang, Yu Wang, Yingchun Li, Zhijian Tang, Sha Dou, Yuanfen Wang, Honglan Zhu, Xue Ye, Tianyu Zhang, Yonghua Zhang, Shufen Li, Yonghong Zhao, Yi Li, Heng Cui, Xiaohong Chang

**Affiliations:** ^1^Department of Obstetrics and Gynecology, Peking University People’s Hospital, Beijing, China; ^2^Center of Gynecologic Oncology, Peking University People’s Hospital, Beijing, China; ^3^Beijing Weixiao Biotechnology Development Limited, Beijing, China; ^4^Department of Radiology, Peking University People’s Hospital, Beijing, China

**Keywords:** ovarian cancer, immunotherapy, adoptive cell therapy, safety and efficiency evaluation, circulating tumor cell

## Abstract

Ovarian cancer is a leading cause of death among gynecological malignancies, and novel therapies are urgently needed. Here we report preliminary findings on the potential safety and efficacy of 6B11-OCIK, an adoptive cell therapy of autologous T cells induced by the humanized anti-idiotypic antibody 6B11 minibody plus dendritic cells and cytokines, against platinum-resistant recurrent or refractory ovarian cancer in three patients. We found that 6B11-OCIK treatment was safe and well tolerated after five cycles of intravenous infusion with an initial dose of 1–2×10^9^ cells and a dose-climbing strategy. Hemoglobin, platelets, white cell count, creatinine or liver enzyme values, coagulation function, kidney and heart function were not significantly affected over the duration of therapy. Two of the three enrolled patients showed potentially drug-related grade 1 and 2 weakness, and no other adverse events were observed. Of the three enrolled patients, one had stable disease and two showed disease progression. The patient with favorable clinical efficacy had better immune response as measured by 6B11-OCIK proliferation capacity, activation ability of CD3+CD8+ tumor-specific cytotoxic T lymphocytes and CD3+CD56+ cytokine-induced killer cells, and tumor cell killing efficiency. Changes in circulating tumor cells after treatment were consistent with serum level CA125 in the patient with stable disease (both decreased), while differences were observed in the two patients with disease progression (increased CA125 in both and decreased CTC in the patient with better immune response), suggesting that variation of circulating tumor cells was more consistent with immune response and reflected efficacy directly. This preliminary study suggested that autologous 6B11-OCIK treatment was safe and had potential clinical efficacy against ovarian cancer. Patients with better immune response had more favorable efficacy. In addition to imaging, CA125 and immunophenotypes, CTC monitoring may represent a potential indicator of immunotherapy response.

## Introduction

Ovarian cancer (OC) has the highest mortality rate among all female reproductive malignancies, with a rate approximately equal to the mortality of cervical cancer and uterine body cancer combined (1). The initial treatment options for epithelial ovarian cancer (EOC) include cytoreductive surgery followed by paclitaxel and platinum chemotherapy (2, 3) or neoadjuvant chemotherapy followed by interval debulking surgery (4). However, most patients with EOC (80%) are diagnosed at late stage. Furthermore, the rate of recurrence for ovarian cancer is high (70%–80%) and patients who develop resistance to frontline therapies have limited treatment options. Therefore, the prognosis for EOC patients remains poor and survival rates have improved only modestly over the past few decades (5). Most patients with EOC die of tumor recurrence and drug resistance. Therefore, identifying new and effective treatment strategies for ovarian cancer patients is critical, particularly for advanced stage patients with platinum-resistant recurrence.

Immunotherapy has been established as an effective treatment for cancer (6–8) and can be applied alone or in combination with other approaches. Immunotherapy can be classified into two categories: (1) active immunotherapy (e.g., cancer vaccines); and (2) passive or adoptive immunotherapy, such as monoclonal antibodies and adoptive cell therapies (ACT). As cellular immunity plays an important role in anti-tumor immunity, ACT has become a powerful treatment strategy for cancers, including ovarian cancer (9, 10). ACT of dendritic cells (DCs), the main antigen presenting cells, together with cytokine induced killer cells (CIKs), has shown great potential to prevent tumor recurrence, increase progression-free survival (PFS) rates, and improve the quality of life of cancer patients (11–14).

In our previous studies (15), we prepared the anti-idiotypic monoclonal antibody 6B11 by immunizing mice with COC166-9, a monoclonal antibody obtained from mice immunized with tissue antigens from ovarian cancer patients. The 6B11 monoclonal antibody mimics the ovarian cancer–associated antigen OC166-9 and induces specific humoral and cellular immunity against ovarian cancer. The humanized modified 6B11 minibody (6B11mini) was obtained by combining the single chain of the 6B11 antibody with the human IgG hinge region (16). Anti-idiotypic 6B11mini–pulsed DCs were shown to induce T cell responses for specific killing against autologous ovarian cancer (17). We thus developed 6B11-OCIK, an ACT strategy against advanced drug-resistant recurrent ovarian cancer. We found that 6B11-OCIK induced not only a large number of specific cytotoxic T lymphocytes (CTLs) amplified by 6B11mini loading DCs, but also non-specific CIKs stimulated with anti-CD3 antibody and cytokines such as IL-2.

Early evaluation of the efficacy of anti-tumor therapy is difficult to conduct because the effects of treatments on overall survival (OS) and PFS require a substantial amount of time for analysis. Currently, the efficacy of drugs on solid tumors is mainly assessed according to Response Evaluation Criteria in Solid Tumors (RECIST v1.1) based on imaging. As the response of immunotherapy is often delayed, conventional imaging evaluation may underestimate the efficacy of immunotherapy in patients with disease progression (18). IrRECIST and iRECIST methods recommended for immunotherapy (19) have not been widely accepted. Therefore, it is necessary to improve the criteria to evaluate the efficacy of immunotherapy for cancer treatment.

Tumor biomarkers and other clinical indicators can evaluate the efficacy of cancer treatments. Circulating tumor cells (CTCs), which are derived from the primary tumor or metastases and play an important role in tumor metastasis, have been widely used as biomarkers for tumor diagnosis and tumor progression through non-invasive and real-time monitoring (20). Recent studies suggested that dynamic changes in CTC numbers may be used to assess the efficacy of cancer treatment (21).

In this study, we performed a preliminary and exploratory study about the safety and efficacy of 6B11-OCIK monotherapy against advanced platinum-resistant recurrent or refractory EOC in three patients. Efficacy of 6B11-OCIK monotherapy was evaluated according to RECIST v1.1 with CT imaging. We evaluated serum biomarker CA125 levels and performed dynamic monitoring of CTC numbers in blood. The potential for CTCs as an indicator of treatment response was evaluated.

## Materials and Methods

### Study Design

The 6B11-OCIK injection strategy is an ACT strategy against stage III–IV platinum-resistant recurrent ovarian cancer. The primary outcome of this study was a safety assessment of 6B11-OCIK through the evaluation of symptoms, vital signs, laboratory and auxiliary tests, adverse events (AE), and severe AEs. The secondary outcome was preliminary efficacy assessed by RECIST based on variation of CT imaging, dynamic changes of CA125 and CTC numbers, and immune response.

### Study Population

Patients diagnosed with stage III–IV platinum-resistant EOC with a maximum measurable lesion smaller than 5 cm in diameter were enrolled in this study. The patients were between 18 and 70 years old, with at least 3 months of expected survival. Inclusion criteria were as follows: White blood cells number >3x10^9^/L, absolute lymphocyte count ≥1.0x10^9^/L, platelet count ≥100x10^9^/L, hemoglobin ≥9 g/dL, Aspertate Aminotransferase and Alanine aminotransferase ≤2.5xULN (for patients with concurrent liver metastasis ≤5xULN), bilirubin ≤1.5xULN (for patients with Gilbert syndrome, bilirubin ≤3xULN), alkaline phosphatase ≤2.5xULN (patients with concurrent liver metastasis ≤5xULN), albumin ≥3 g/dL, serum creatinine and/or urea <1.5 times normal, prothrombin time: INR < 1.7 or prothrombin extension time < 4 sec, and ECOG ≤1. Exclusion criteria were as follows: central nervous system metastasis or active central nervous system injury, corticosteroids or other systemic immunotherapy within 4 weeks, interstitial lung disease or interstitial pneumonia, autoimmune diseases, pregnancy or lactation, other malignancies, uncontrolled concomitant diseases, active infectious diseases, severe allergic disorders, and previous gene therapy or other lymphocyte-based immunotherapy. Participants who were receiving or had previously received systemic therapy for any other malignancy in the preceding 4 weeks were also ineligible. Three patients were enrolled in this study.

### Expansion of 6B11-OCIK and Treatment Protocol

Peripheral blood of patients was collected (blood collection volume (ml) = 1-2*10^8^/absolute value of lymphocytes per ml). Peripheral blood mononuclear cells (PBMNCs) were isolated by Ficoll density gradient centrifugation and then transferred to serum-free lymphocyte culture medium supplemented with 1000 U/ml IL-4, 1000 U/ml GM-CSF, and 5 μg/ml 6B11 minibody in a T75 cell culture flask. The cells were incubated at 37°C in a CO_2_ incubator for 48 h. Adherent cells and cells in suspension were collected and transferred to a T225 activated culture flask coated with anti-human CD3 antibody for co-culture. Serum-free lymphocyte culture medium containing 500IU/ml human recombinant interleukin-2 (IL-2) was added for subsequent generation and amplification, and cells were harvested within 10–27 days.

Cell infusion was performed five times (at approximately day 1 (D1), D6, D11, D25 and D39). 6B11-OCIK for the 1st - 3rd infusion was prepared by once collection of peripheral blood, and for the fourth and fifth time, sufficient peripheral blood was collected respectively. Each patient received 1–2×10^9^ cells as the initial cell infusion dose on day 1, followed by 3–5×10^9^ cells on day 6 ± 2, 6–10×10^9^ cells on day 11 ± 2, and 6–10×10^9^ cells on days 25 ± 7 and 39 ± 7. The maximum dose was 10×10^9^ cells. If the cell culture failed to reach the specified number of cells (6–10×10^9^) during the third, fourth and fifth cell infusion, the patients were transfused with the maximum number of cells obtained in the actual culture.

### Characterization of 6B11-OCIK Cells

(1) Cell proliferation detection: The cell numbers and survival rates of 6B11-OCIK before infusion were tested. Cell proliferation was evaluated and compared with PBMNCs before culture.
(2) Analysis of DC activation: The phenotypes of antigen-presenting DCs, including CD86, CD80, CD1a, CD83, HLA-DR CD54, and CD40 expressions, were identified in fresh isolated PBMNCs and 6B11-OCIK by flow cytometry.
(3) Detection of lymphocyte activation: The changes in lymphocyte cell populations were detected by flow cytometry in PBMNCs and 6B11-OCIK, including CD3+ lymphocytes, CD3+CD4+ helper T cells, CD3+CD8+ killer T cells, CD3-CD56+ NK cells and CD3+CD56+ NK-like T cells.
(4) Anti-tumor function of 6B11-OCIK: The killing effect of 6B11-OCIK *in vitro* was evaluated using the tumor cell line HOC1A (effect-target ratios, 10:1, 25:1, 50:1; treatment for 4 h) and a real-time cell analysis instrument (ACEA Biosciences, Inc.).

### Detection of CTCs

6 ml peripheral blood from patients was collected into an ACD anticoagulant tube (Becton Dickinson, Franklin Lakes, NJ, USA) at D0 (before the first treatment), D25 (after the third treatment), and D50 (after the last treatment). Samples were stored at room temperature and in dark for no more than 48 h prior to processing. Detection of CTCs were performed as protocols described in our previous study (22), and briefly as the following procedures.

(1) Subtraction enrichment of CTCs: Subtraction enrichment was performed using SE kit (Cytelligen, San Diego, CA, USA) according to the manufacturer’s instructions. Blood samples were centrifuged at 200 g for 15 min at room temperature. The cell pellet was gently resuspended with 3.5 mL of CRC buffer, followed by slow loading on a 3-ml cell separation matrix in a 50-mL tube and subsequent centrifugation at 350 g for 6 min to remove red blood cells. The solution containing WBCs and tumor cells was collected into a 50-mL tube and incubated with 300 μl of immune-magnetic beads conjugated to a cocktail of anti-leukocyte mAbs at room temperature for 20 min with gentle shaking. WBCs bound to immune-magnetic beads were depleted using a magnetic separator. The remaining solution was collected into a 50-mL tube, followed by the addition of CRC buffer and centrifugation at 500 g for 5 min at room temperature. The supernatant was discarded and the cell pellet containing CTCs was gently resuspended in 100 µl residual liquid for subsequent analysis.
(2) Immunofluorescence and fluorescence *in situ* hybridization (iFISH) identification of CTCs: CTCs in the enriched cells were identified by iFISH (Cytelligen) according to the manufacturer’s instructions with some changes. Briefly, the enriched cells containing CTCs in 100 μl CRC buffer were gently mixed with 2 µl antigen repair buffer at room temperature for 10 min. Samples were subsequently incubated with 200 μl of an immunofluorescence staining mixture of mAbs recognizing HE4, CA125, CD45 and CD31 conjugated to Alexa Fluor (AF) 488, CY7, AF 594, and CY5, respectively, at room temperature for 20 min in the dark. After washing, samples were mixed with 100 μl cell fixative and applied onto formatted and coated slides to dry in an oven at 30–32°C overnight. Air-dried slides were re-fixed by cell fixative and FISH analysis was performed with a chromosome 8 centromere probe (CEP8) (Abbott Laboratories, Spectrum Orange) for 4 h using an S500 Stat Spin Thermo Brite Slide Hybridization/Denaturation System (Abbott Molecular). Samples were mounted with mounting media containing DAPI (Vector Laboratories) for nucleus staining and subjected to automated CTC image scanning and analyses by the fully automated scanning and image analyzing system, Metafer-iFISH (CarlZeiss, MetaSystems, and Cytelligen). CTCs of ovarian cancer were identified as DAPI+/CD45-/CD31-/HE4+ or CA125+ or DAPI+/CD45−/CD31- with aneuploidy of chromosome 8.

### Detection of the CA125 Tumor Biomarker

Serum CA125 levels were detected by enzyme linked immunosorbent assay (Roche Diagnostic, Germany) at D0 (before the first treatment), D25 (after the third treatment), and D50 (after the last treatment).

### Safety Assessment

The safety of 6B11-OCIK was determined by monitoring patients from day 1 to day 67 (approximately 4 weeks after the last cell infusion). We evaluated symptoms, vital signs, laboratory and auxiliary tests, AEs, and severe AEs.

### Tumor Progression Assessment

Chest, abdomen, and pelvic enhanced CT scans were obtained before the first treatment (D0) and after the fifth treatment (D50) of 6B11-OCIK. Brain MRI was performed if necessary. Tumor progression assessment was analyzed using RECIST version 1.1 and described as complete remission (CR), partial remission (PR), progressive disease (PD) and stable disease (SD).

### Immune Monitoring of Patients

Flow cytometry was performed on peripheral venous blood from patients to measure the change in immune cell populations, including CD3+ lymphocytes, CD3+CD4+ helper T cells, CD3+CD8+ killer T cells, CD3-CD56+ NK cells, and CD3-CD19+ B cells before treatment and after each administration of 6B11-OCIK.

### Statistical Analysis

Analyses for the demographic and clinical features were descriptive. The paired t test was used to compare the percentage and surface biomarker expression of immune cell subsets before and after therapy. The unpaired t test was used to compare the differences between patients. A P value <0.05 was considered statistically significant. Statistical analyses were performed using GraphPad Prism software (GraphPad Software Inc.).

## Results

### Study Population

Three eligible patients were enrolled from March 2018 to August 2019. The characteristics of the enrolled patients are listed in **Table 1**. All three patients underwent multiple lines of treatment and had platinum-resistant recurrent or refractory EOC. Patient 1 (6B11-OCIK-001; high-grade serous carcinoma stage IIIC) had received five lines chemotherapy in 2 years after the first ovarian cancer cytoreductive surgery. Patient 2 (6B11-OCIK-002; high-grade serous carcinoma stage IIIC) received standard first-line chemotherapy with paclitaxel and carboplatin after the first ovary tumor debulking operation. Patient 3 (6B11-OCIK-003; high-grade serous carcinoma stage IIIB) underwent cytoreductive operations three times followed by first to three-line chemotherapies. Serum CA125 levels of all three patients were instable or even increasing. CT revealed that all patients had a maximum measurable lesion of smaller than 5 cm in diameter. Pathology imaging of the tumors of the three patients is shown in **Supplementary Figure S1**.

**Table 1:** Characteristics of the enrolled patients.

Case NO.	Age	Operation date	Pathological diagnosis	Chemotherapy regimens after operation	*serum CA125 before 6B11-OCIK treatment (U/ml)	CT image of patients before 6B11-OCIK treatment	6B11-OCIK treatment(D1, D6, D11, D25, D39)
1	44	1/5/2016	High-grade serous carcinoma stage IIIC	2/2016-6/2017: 3-step chemotherapy: liposome paclitaxel + loplatin *7; Cyclophosphamide + etoposide *6; Cyclophosphamide + carboplatin *2. 6/2017: Recurred with drug-resistant 8/2017-10/2017: Carboplatin + paclitaxel *4 11/2017-3/2018: gemcitabine + bevacizumab (28 days therapy in weeks 1、8、15) 5/2018: albumin paclitaxel + bevacizumab weekly therapy *3	Before initial operation: 815 8/15/2016:322 8/21/2017:117 10/30/2017:566 3/8/2018:122	Low-density nodules at the posterior margin of segment S6 of the liver and multiple intrahepatic nodules were considered as metastatic tumors. Multiple nodular peritoneum thickening suggested implantation metastasis. Lymph nodes were found in abdominal pelvic cavity and bilateral iliac, among which multiple enlarged were found near the right iliac vessels, and lymphatic metastasis was considered.	8/16/2018 8/22/2018 8/29/2018 9/17/2018 9/30/2018
2	55	2/23/2018	High-grade serous carcinoma stage IIIC	2/2018-7/2018: paclitaxel liposome (240mg) + carboplatin(500mg) *5; paclitaxel (210mg) + carboplatin (500mg).	Before initial operation:5000+ 9/25/2018:34.52 10/24/2018:91.61 11/17/2018:161.2	Multiple small lymph nodes were found in pelvic cavity and retroperitoneum; Splenic tubercle*; Left adrenal gland with poor shape*	11/28/2018 12/3/2018 12/7/2018 12/28/2018 1/9/2019
3	52	9/24/2014 11/29/2016 4/24/2018	Stage IIIB of high-grade serous adenocarcinoma	10/2014-5/2015: Paclitaxel + carboplatin *8; 12/2016-5/2017:TC*3, taxol (240mg) +eloxatin (200mg) *1, IAP*2; 5/2018-12/2018:paclitaxel Liposome(150-210mg)*10+ Arsenious acid and sodium chloride injection (10mg) (7-10 daily a month*8 months); 12/2018: fluorouracil(1.45g) *3; Doxorubicin *1(60mg)	Before initial operation:338.2 3/2/2018:127.6 3/29/2018:239.4 5/14/2018:308.5	Multiple enlarged lymph nodes were found near the abdominal aorta and left iliac vessels. Metastatic tumors were considered	6/17/2019 6/21/2019 6/26/2019 7/8/2019 7/18/2019

*Normal serum CA125 level was ≤35 U/ml.

### Evaluation of Safety and Tolerance

Five times cell infusion were performed (at approximately day 1 (D1), D6, D11, D25 and D39). The treatment of 6B11-OCIK intravenous infusion for the three patients with platinum-resistant recurrent or refractory EOC was safe and well tolerated. We observed that 6B11-OCIK infusions did not significantly affect hemoglobin, platelets, white cell count, creatinine or liver enzyme values, coagulation function, kidney function and heart function over the duration of therapy. Two patients showed grade 1 and 2 weakness that might be drug-related, and no other AEs were found (**Table 2**).

**Table 2:** Observed adverse events.

Patient	Adverse Event	DLT	Severity	SAE	Relationship	Measure 1	Measure 2	Outcome
001	transient chest pain(2-3S)	NO	1	NO	4	2	1	1
	abdominal distension	NO	1	NO	3	2	1	1
	weakness	NO	1	NO	4	2	1	3
	abdominal distension worsened	NO	2	NO	4	6	1	3
	waist up worsened	NO	2	NO	4	6	1	3
	pelvic pain worsened	NO	2	NO	4	6	1	3
	abdominal burning sensation	NO	2	NO	4	6	1	3
	nausea	NO	1	NO	4	6	2	3
	vomiting	NO	1	NO	4	6	2	3
	weak	NO	2	NO	2	2	1	1
002	herpes simplex	NO	1	NO	4	2	2	1
	lymphocytopenia	NO	1	NO	3	2	1	1
	elevated urine bacteria	NO	1	NO	4	2	1	1
	weakness	NO	1	NO	2	2	1	1
	elevated urine bacteria	NO	1	NO	4	2	1	1
	elevated urinary leukocyte	NO	1	NO	4	2	1	1
	fever	NO	1	NO	3	2	1	1
	abdominal pain	NO	1	NO	4	6	1	1
003	noninfectious diarrhea	NO	1	NO	4	2	2	1

(DLT and SAE were not observed).

DLT, dose limiting toxicity.

Severity: 1=Grade 1; 2=Grade 2; 3=Grade 3; 4=Grade 4; 5=Grade 5.

SAE, severe adverse event.

Relationship, the relationship with research drugs. 1=yes; 2=may be 2; 3=may not be; 4=sure not be; 5= unable to determine.

Measure 1: Measures taken with respect to experimental drugs. 1=dose increase; 2= same dose; 3=dose decrease; 4=suspended medication; 5= termination of medication; 6= inapplicability; 7=unknown.

Measure 2: Measures taken with respect to patients. 1=none; 2=drug combination; 3=therapy combination; 4=quit the test; 5=others.

Outcome: 1=recovery/cure; 2=recovery/cure with sequelae; 3=recovering/improving; 4=quit the test; 5=others.

### Evaluation of Tumor Progression After 6B11-OCIK Treatment

**Figure 1:**
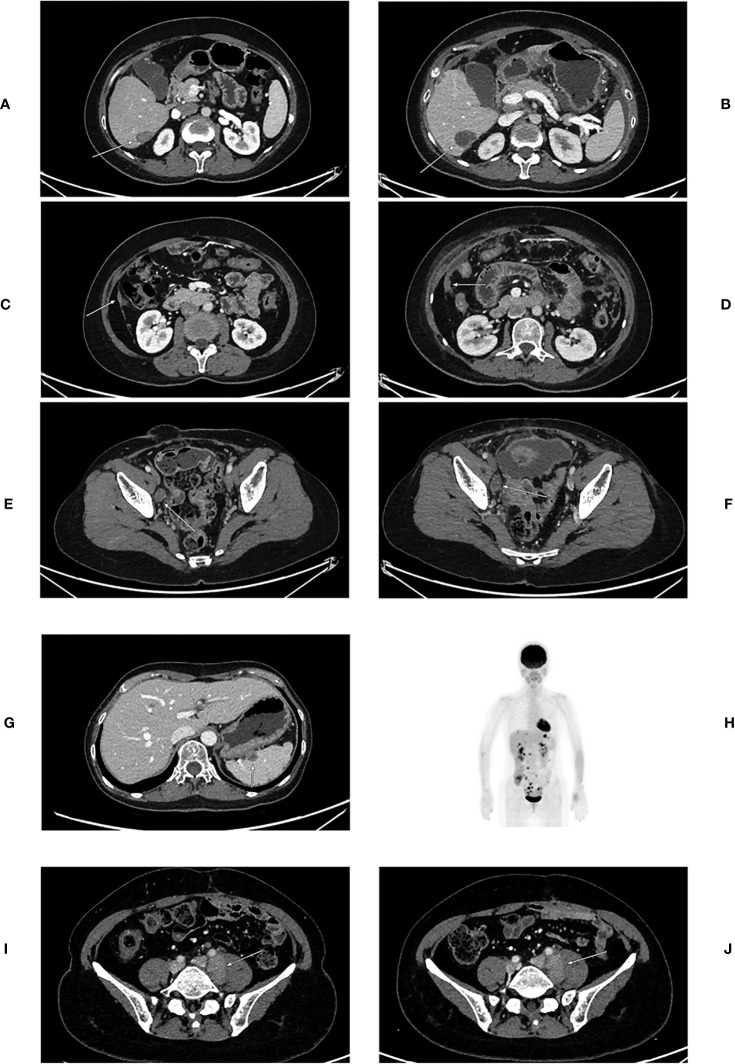
CT images of patients before and after 6B11-OCIK treatment. **(A)** Lesion 1 of patient 1 before 6B11-CIK treatment: Plain and contrast-enhanced computed tomography shows a low-density lesion in S6 segment of liver, with slight enhancement. **(B)** Lesion 1 of patient 1 after 6B11-CIK treatment: CT shows that the lesion in S6 segment of liver became enlarged. **(C)** Lesion 2 of patient 1 before 6B11-CIK treatment: CT shows local peritoneal thickening. **(D)** Lesion 2 of patient 1 after 6B11-CIK treatment: Local peritoneum became thicker, and peritoneal effusion was detected. **(E)** Lesion 3 of patient 1 before 6B11-CIK treatment: Right iliac perivascular lymph node enlargement before treatment. **(F)** Lesion 3 of patient 1 after 6B11-CIK treatment: The lymph nodes near the right iliac vessels were slightly enlarged; pelvic effusion was detected. **(G)** Lesions of patient 2 before 6B11-CIK treatment: Low-density nodules were observed in the spleen. **(H)** PET-CT of patient 2 after 6B11-CIK treatment: Multiple FDG metabolism enhancement lesions were found throughout the body, and tumor recurrence and metastasis were considered. The lesions involved the right adrenal gland, liver capsule, spleen capsule, peritoneum, intestinal surface and multiple lymph nodes at heart diaphragm angle, left costal phrenic angle, mesenteric, abdominal aorta and iliac vascular periphery. **(I)** Lesions of patient 3 before 6B11-CIK treatment: Enlarged lymph nodes near the left iliac vessels were observed with a size of approximately 3.6×2.2cm. **(J)** Lesions of patient 3 after 6B11-CIK treatment: Left iliac perivascular enlarged lymph nodes with no change after treatment.

RECIST (V1.1) was used to evaluate the tumor progression of the three patients according to CT imaging (**Table 3** and **Figure 1**). Patients 1 and 2 showed PD, including enlargement of the original metastatic lesion and appearance of new metastatic lesions. Patient 3 showed SD with no significant change in the original metastatic lesion before and after treatment.

**Table 3:** Evaluation of tumor/progression before and after 6B11-OCIK treatment.

Case NO.	CT image of patients before 6B11-OCIK treatment	CT image of patients after 6B11-OCIK treatment	Tumor progression assessment (RECIST v 1.1)
1	Low-density nodules at the posterior margin of segment S6 of the liver and multiple intrahepatic nodules were considered as metastatic tumors. Multiple nodular peritoneum thickening suggested implantation metastasis. Lymph nodes were found in abdominal pelvic cavity and bilateral iliac, among which multiple enlarged nodes were found near the right iliac vessels, and lymphatic metastasis was considered.	Metastatic tumor progression (PD) was considered because nodules at the posterior margin of segment S6 of the liver were fused and enlarged, and some intrahepatic nodules were slightly enlarged. Implantation metastasis of multiple nodular peritoneum thickening were thicker than before. Multiple enlarged lymph nodes in abdominal pelvic cavity and retroperitoneal, considering lymphatic metastasis, increased in number and became larger. Multiple abdominal and pelvic effusions and left pleural effusion were found.	PD
2	Multiple small lymph nodes were found in pelvic cavity and retroperitoneum; splenic tubercle*; Left adrenal gland with poor shape*	(PET-CT) Multiple FDG metabolism enhancement lesions were found throughout the body, and tumor recurrence and metastasis were considered. The lesions involved the right adrenal gland, liver capsule, spleen capsule, peritoneum and intestinal surface, and multiple lymph nodes at heart diaphragm angle, left costal phrenic angle, mesenteric, abdominal aorta and iliac vascular periphery.	PD
3	Multiple enlarged lymph nodes were found near the abdominal aorta and left iliac vessels. Metastatic tumors were considered	Multiple enlarged lymph nodes were found near the abdominal aorta and left iliac vessels. Metastatic tumors were considered, but there was no significant change compared with before treatment.	SD

*Suspected.

RECIST, Response Evaluation Criteria in Solid Tumors; PD, progressive disease; SD, stable disease.

### Changes in Serum CA125 Level After 6B11-OCIK Treatment

**Figure 2:**
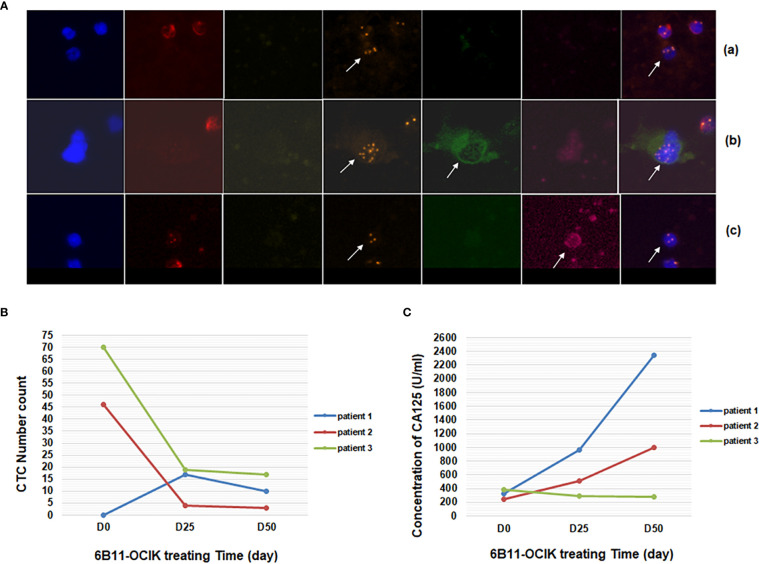
Detection of CTCs and serum CA125 during 6B11-OCIK treatment. **(A)** Identification of CTCs by iFISH: CTCs of ovarian cancer were pointed by arrows. (a): DAPI+/CD45-/CD31- with aneuploidy of chromosome 8; (b): DAPI+/CD45-/CD31-/HE4+; (c): DAPI+/CD45-/CD31-/CA125+. **(B)** Variation of the number of CTCs during 6B11-OCIK treatment: During the treatment of 6B11-OCIK, the number of CTCs increased in patient 1 (from 0 to 17 to 10) and decreased in patient 2 (from 46 to 4 to 3) and patient 3 (from 70 to 19 to 17) during the treatment. **(C)** Variation of serum CA125 during 6B11-OCIK treatment: During the treatment of 6B11-OCIK, CA125 levels in patient 1 and patient 2 increased. The increase was higher in patient 1 (from 324.3 to 2347 U/ml) compared with patient 2 (from 247.6 to 994 U/ml). CA125 levels in patient 3 decreased from 380.4 to 283.5 U/ml.

As shown in **Figure 2C** and **Supplementary Table S1**, during the treatment of 6B11-OCIK, CA125 levels in patient 1 and patient 2 increased, and the increase was higher in patient 1 (from 324.3 to 2347 U/ml) compared with patient 2 (from 247.6 to 994 U/ml). CA125 levels in patient 3 decreased from 380.4 to 283.5 U/ml. Variations of CA125 levels in the three patients were consistent with the tumor progression assessment of the patients.

### Variation of CTC Numbers During 6B11-OCIK Treatment

Cells negative for vascular endothelial cell and leukocyte biomarkers (CD31- CD45-) and with positive expression of tumor biomarkers (HE4+ or CA125+) or chromosome 8 aneuploidy were defined as CTCs of ovarian cancer (DAPI+/CD45-/CD31-/HE4+ or CA125+, or DAPI+/CD45-/CD31- with aneuploidy of chromosome 8) (**Figure 2A**) (manuscript submitted). Chromosome 8 aneuploidy was an important identification character of CTCs. The most prevalent aneuploidy for chromosome 8 of CTCs was pentaploid and above, followed by triploid, tetraploid, and haploid (**Supplementary Figure S2**). The number of CTCs increased in patient 1 (from 0 to 17 to 10) and decreased in patient 2 (from 46 to 4 to 3) and patient 3 (from 70 to 19 to 17) during the treatment (**Figure 2B** and **Table S2**). The variation of CTCs was consistent with tumor progression assessment and CA125 variation in patient 1 (increased CTCs and CA125, PD) and patient 3 (decreased CTCs and CA125, SD), while differences were observed in patient 2 (decreased CTCs and increased CA125, PD) (**Figures 2B, C**). As shown in **Table S2**, tumor biomarker proteins HE4 and CA125 were only expressed in some cells with chromosome aneuploidy. These biomarker-positive cells did not increase the CTC numbers because CTCs were mostly counted by chromosome aneuploidy. Circulating tumor microemboli were detected in patients before and during treatment. Small cell CTCs are usually missed in cell size separation methods, but were captured here by the selective enrichment method and accounted for a large proportion of CTCs. The change of small cell CTC numbers in each patient after treatment was consistent with the change of the total number of CTCs (**Table S2** and **Figure 2B**).

### Characterization of 6B11-OCIK Cells

**Figure 3:**
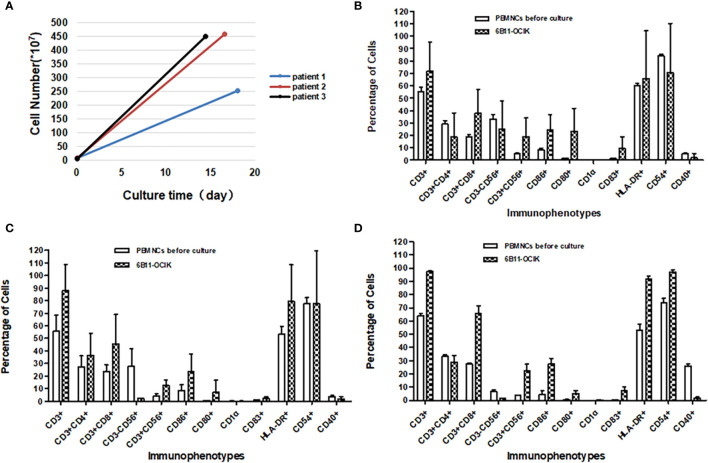
Amplification and activation characterization of ex vivo–expanded 6B11-OCIK cells. **(A)** Proliferation of 6B11-OCIK during culture: PBMNCs were amplified in all three patients, with the average cell amplification of 46.92-fold (patient 1), 102.07-fold (patient 2) and 117.95-fold (patient 3). **(B-D)** Immunophenotypic analysis of expanded 6B11-OCIK and PBMNCs before culture in patients. **(B)** patient 1; **(C)** patient 2; **(D)** patient 3. Results showed activation of DCs (CD86, CD80, CD83, and HLA- DR positive) in 6B11-OCIK. The proportion of CD3+ T lymphocytes, specific CD3+CD8+ killer T cells (CTLs), and CD3+CD56+ NK-like T cells (CIKs) in 6B11-OCIK of all three patients were markedly increased. The T cell proliferation and activation of patient 3 was greater than that of patient 2 and patient 1.

Peripheral blood was collected from each patient at three time points as described in Methods, and PBMNCs were isolated to culture and induce 6B11-OCIK. DCs were first activated by IL-4 and GM-CSF, and specific CTLs were expanded by 6B11 minibody loading DCs. CIKs were stimulated by anti-CD3 and IL-2. The average cell culture days for the three patients were 18, 16.6 and 14.4 days. The average PBMNC amplification was 46.92-fold (patient 1), 102.07-fold (patient 2) and 117.95-fold (patient 3) (**Figure 3A** and **Table S3**).

Immunophenotypic analysis was performed on expanded 6B11-OCIK compared with PBMNCs before culture. The results showed activation of DCs (CD86, CD80, CD83, and HLA-DR positive) in 6B11-OCIK. Activation of CD54+ DCs in patient 3 was also observed. There was no activation of CD54+ DCs in patients 1 and 2, which could be attributed to the poor cell quality in batch 4, as the activation of CD54+ DCs was apparent in patients 1 and 2 for other batches (**Figures 3B–D** and **Tables S4**–**7**).

The proportions of CD3+ T lymphocytes, specific CD3+CD8+ killer T cells (CTLs), and CD3+CD56+ NK-like T cells (CIKs) in 6B11-OCIK of all three patients were markedly increased. The proportion of CD3+CD4+ helper T cells in patient 2 was also increased, and this population may help to activate CD3+CD8+ killer T cells. Proliferation and activation of T cells from patient 3 were greater than that of patient 1 and patient 2 (**Figures 3B–D** and **Tables S4**–**7**).

**Figure 4:**
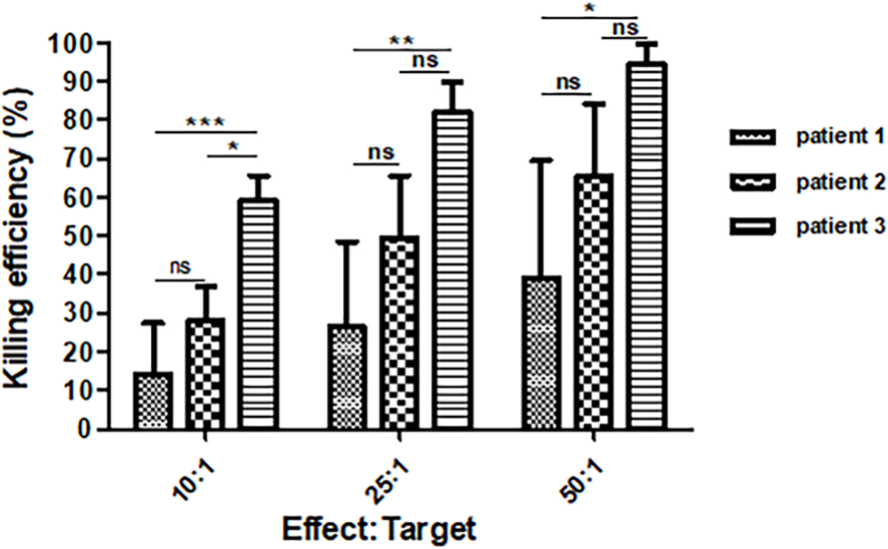
Killing efficiency of 6B11-OCIK against the ovarian cancer cell line HOC1A. The average killing efficiency of 6B11-OCIK from all three patients against the ovarian cancer cell line HOC1A increased with the increase of effect-target ratio. At each effect-target ratio, the killing efficiency was the lowest for patient 1 and highest for patient 3. *P < 0.05; **P < 0.01; ***P < 0.001; ns, no significance, P > 0.05.

If the decreased CTCs were due to 6B11-OCIK, the tumor killing function of 6B11-OCIK *in vitro* may reflect its potential clinical efficacy. We found that 6B11-OCIK of the three patients effectively killed HOC1A ovarian cancer cells, and the killing efficiency increased with the increase of the effect-target ratio. The killing efficiency was the lowest for patient 1 and highest for patient 3 (**Table S8**, **Figures 4** and **S3**).

Together these results indicate that in terms of cell amplification, activation of DCs, CTLs and CIKs, and tumor killing function, patient 3 had a better response than patient 2, who in turn showed better response than patient 1. These results are also consistent with the increased CTCs in patient 1 but decreased CTCs in patient 2.

### Changes of Peripheral Blood Lymphocyte Phenotypes in Patients During 6B11-OCIK Treatment

As shown in **Supplementary Figure S4**, during the treatment of 6B11-OCIK, the proportion of lymphocytes of patient 1 and patient 2 barely changed, while the proportion of CD4+ and CD8+ T cells of patient 3 increased, suggesting improvement of the overall immune function of patient 3 *in vivo*.

### Summary of the Clinical Feature Changes After 6B11-OCIK Treatment

The results of clinical characteristics after 6B11-OCIK treatment are shown in **Table 4**. After 6B11-OCIK treatment, patient 3 had SD and decreased CA125 and CTCs, while patients 1 and 2 with PD showed increased CA125 as well as increased CTCs in patient 1 and decreased CTCs in patient 2. The activation of DCs (CD86 +, CD80+, CD83+, HLA-DR+), CTLs (CD3+CD8+) and NKT (CD3+CD56+) cells were all increased in 6B11-OCIK of the three patients during *in vitro* culture and showed good killing effect on ovarian cancer cells. The killing effect of 6B11-OCIK in patient 3 was better than for 6B11-OCIK in patient 2, and the killing effect of 6B11-OCIK in patient 2 was better than for 6B11-OCIK in patient 1. After 6B11-OCIK treatment, the immune function of patient 3 was improved *in vivo*, and the ratios of CD3+CD4+ and CD3+CD8+ were increased to a certain extent.

**Table 4:** Summary of the clinical feature changes after 6B11-OCIK treatment.

Case NO.	Tumor progression assessment (RECISTv 1.1)	Variation of CA125 (U/ml)	Variation of CTC	Proliferation ratio of lymphocyte culture	Activation of DC in 6B11-OCIK	Activation of lymphocyte in 6B11-OCIK	Killing effect(10:1; 25:1; 50:1)	immune function in *vivo*	Follow up(4/12/2020; 6/24/2021)
1	PD	Increase 324.3 958.2 2347	Increase 0 17 10	46.92	increase CD86+ CD80+ CD83+ HLA-DR+	increase CD3+, CD3+CD8+ CD3+CD56+	14.32% 26.56% 39.22%		CA125 continued to rise beyond the detection range, and intestinal obstruction occurred. Try of oral etoposide and other chemotherapy regiments showed no obvious effect. Try of intestinal obstruction surgery failed, attempts of abdominal surgery failed (frozen abdominal cavity), and the patient died on March 6, 2019(about 5 months after the last 6B11-OCIK treatment)
2	PD	Increase 247.6 506.1 994	decrease 46 4 3	102.07	increase CD86+ CD80+ CD83+ HLA-DR+	increase CD3+, CD3+CD4+ CD3+CD8+ CD3+CD56+	28.08% 49.51% 65.33%		After treatment, the patient were sensitive to chemotherapy(cisplatin + gemcitabine + Bevacizumab *3, Oxaliplatin + gemcitabine + Bevacizumab *3),the CA125 decreased to normal, and the lesions were reduced, then under Bevacizumab maintenance therapy.CA125 continued to increase and the lesion reappeared a few months later. CA125 growth slowed with maintenance treatment of Olaparib, but increased again after discontinuation of treatment. In 2020, the patients were treated with lenvatinib + Capecitabine for 4-5 months, and the growth of CA125 was moderate, the lesion was slightly smaller, but the hand-foot syndrome was obvious and the pulmonary fluid was fluid. CA125 and lesions continued to increase after drug withdrawal. Then maintenance therapy with lenvatinib + Niraparib. Due to multi-line drug resistance, large side effect, Lung metastasis and compression of heart failure, the patient died in12/15/2020(nearly two year after the last 6B11-OCIK treatment.).
3	SD	Decrease 380.4 292.1 283.5	decrease 70 19 17	117.95	increase CD86+ CD80+ CD83+ HLA-DR+	increase CD3+ CD3+CD8+ CD3+CD56+	59.12% 82.08% 94.26%	increase: CD3+CD4+ CD3+CD8+	Satisfactory tumor cell reduction was performed on 9/16/2019; The patient was sensitive to chemotherapy (albumin paclitaxel 400+ carboplatin 600*4), CA125 decreased (49.94). (2/14/2020) IAP (D1-2, oxalate platinum 180mg D1, liposomal doxorubicin 40mg D2). Due to the development of drug resistance and the discovery of tumor foci in iliac artery, targeted drug maintenance therapy: Olaparib *2; Lenvatinib + Olaparib *3, CA125 28.85 (11/6/2020). The patient is in good condition with few side effects. Physical examination found mediastinal lymphatic metastasis in the chest and tumor foci in iliac artery larger (4/2021). Target drug was stopped and gemcitabine + Lenvatinib was used. First course of CA125 decreased (170-) She is currently undergoing the second course of treatment. (survive)

RECIST, Response Evaluation Criteria in Solid Tumors; PD, progressive disease; SD, stable disease.

## Discussion

Up to 85% of patients with advanced ovarian cancer show recurrence after standard therapy of a combination of debulking surgery and platinum-based chemotherapy (1). Therefore, identifying new treatments for these patients is critical. Here we performed a preliminary evaluation of the safety and efficacy of 6B11-OCIK as potential monotherapy against platinum-resistant recurrent or refractory EOC. All three participants had relapsed EOC after multiple lines of treatment, based on CT imaging and abnormal serum CA125 level (>30 U/ml). After five cycles of 6B11-OCIK cell transfusion, two patients showed grade 1 and 2 weakness that might be drug-related; no other treatment-related adverse reactions were found. While tumor progression was observed in two patients, tumor progression remained stable in one patient. This suggested that even in patients with advanced platinum-resistant recurrent or refractory EOC, and even as monotherapy, 6B11-OCIK may control tumor progression in some patients.

Changes of tumor biomarkers and other clinical measures can be used to evaluate efficacy of cancer treatments. In this study, the changes in the levels of the cancer biomarker CA125 were consistent with tumor progression as determined by imaging. CA125 serum levels were increased in the two PD patients and decreased in the patient with SD. However, because of the long-term presence of a large tumor load in patients with advanced stage cancers, the change of CA125 levels might reflect the large tumor load but not directly reflect the dynamics of tumor cell invasion and metastasis. Therefore, a dynamic monitoring method to directly and objectively determine the efficacy of immunotherapy is required.

CTCs are a novel tumor biomarker that have been approved by the US Food and Drug Administration for monitoring breast cancer, colorectal cancer, prostate cancer and other solid tumors and play an important role in tumor diagnosis and prognosis (23, 24). The value of CTCs as an indicator of efficacy of cancer treatments was recently confirmed. An analysis of five phase 3 clinical trials in prostate cancer demonstrated that CTC number was a far better measure of treatment response compared with prostate serum antigen, the current standard biomarker for prostate cancer (21).

Intra-abdominal implantation metastasis was previously considered the main route of ovarian cancer metastasis, and blood metastasis was thought to be less important (25). However, Pradeep et al. showed that blood metastasis was important for ovarian cancer metastasis (including proximal omentum metastasis), because in a mouse model of parabiosis with shared blood circulation system, no matter inoculated ovarian cancer cells in the peritoneal cavity or *in situ* ovary of host mice, tumor metastases occurred in the omentum of the symbiotic host mice (26). Increasing studies have since confirmed the presence of CTCs in the peripheral blood of ovarian cancer patients and their potential use as a tumor biomarker for diagnosis, treatment response, prognosis, and recurrence and metastasis monitoring of ovarian cancer (27–30).

In this study, in addition to conventional clinical methods to assess drug efficacy, such as RECIST v1.1 based on imaging and the tumor biomarker CA125, we also evaluated the efficacy of 6B11-OCIK therapy by dynamically monitoring changes in patient CTCs using SE-iFISH, a new method for CTC detection (31, 32). CTC monitoring by SE-iFISH has shown high sensitivity and specificity in gastric cancer, colon cancer, liver cancer, and other cancers (33–35). In our previous study, CTC detection by SE-iFISH has also shown good diagnostic value in ovarian cancer (22). In the SE-iFISH method, CTCs are separated by subtraction enrichment, in which combinations of multiple antibodies including anti-CD45 are used to remove WBCs and enrich CTCs. Compared with other CTC enrichment strategies such as positive enrichment and molecular sieve methods, subtraction enrichment is not restricted by tumor antigen expression, epithelial mesenchymal transformation and cell size, and this strategy can obtain highly heterogeneous CTCs. Then in the process of CTC identification, in addition to immunofluorescence detection of tumor cell surface molecules, chromosome 8 aneuploidy, a common phenomenon in various tumors, was also evaluated by fluorescence in-situ hybridization. This approach tracks CTCs with positive expression of tumor biomarkers or/and chromosomal heteroploidy.

In this study, the number of CTCs in the patient with SD decreased (from 70 to 19 to 17) after 6B11-OCIK treatment, suggesting that 6B11-OCIK may suppress tumor cell growth in the blood. In the two PD patients, CTC numbers increased in one case (from 0 to 17 to 10) and decreased in the other patient (from 46 to 4 to 3), indicating that 6B11-OCIK may reduce tumor cell growth and slow tumor metastasis. However, local tumor progression was not halted due to limited efficacy or delayed effect.

The immune function of patients could also be used as an important indicator to predict or assess the overall efficacy of cellular immunotherapy. Our results showed that the proliferation and activation capacity of 6B11-OCIK *in vitro* reflected the immune status of patients. We analyzed the possible relationship between the characteristics of 6B11-OCIK cells and clinical efficacy. In terms of cell amplification ability, activation of DCs, CTLs and CIKs, and *in vitro* tumor killing by 6B11-OCIK, patient 3 had a better response than patient 2 and patient 2 had a better response than patient 1. This was consistent with the clinical efficacy: patient 3 had the best response (SD, decreased CA125 and CTCs), followed by patient 2 (PD, increased CA125, but decreased CTCs); patient 1 had poor response (PD, increased CA125 and CTCs). The different immune response between patients 1 and 2 (the two PD patients) might also explain the differences in CTC number changes (increased in patient 1 and decreased in patient 2).

The anti-tumor response is dominated by T-lymphocyte-mediated cellular immune response. Therefore, changes in T-lymphocyte subsets can better reflect the cellular immune function (36). In this study, the proportions of CD3+ T lymphocytes, CD3+CD8+ killer T cells (CTLs), and CD3+CD56+ NK-like T cells (CIKs) in all three patients were remarkably increased after treatment, and the proportion of CD3+CD4+ helper T cells in patient 2 was also increased. In 6B11-OCIK, CTLs are amplified by 6B11mini-loaded DCs, and CIK cells are simultaneously induced by anti-CD3 antibody and cytokines such as IL-2, two main effectors against tumors. The main effectors of CIK cells are the NK-like T lymphocytes (CD3+ CD56+) that have potentially enhanced and broad antitumor activity and do not depend on TCR and MHC activity but still can elicit both MHC-restricted and MHC-unrestricted anti-tumor cytotoxicity (7, 37). CIKs may be useful in the adjuvant therapy of postoperative chemotherapy in EOC patients (38). CD8+ T lymphocytes play an important role in anti-tumor immune response. After antigen activation, they recognize specific antigens and play an important role in the direct killing of tumor cells by releasing effector molecules such as perforin and granulase. Previous studies showed that CD8+ T cells improved immune surveillance, prognosis and survival in a murine ovarian cancer model (39, 40). CD4+T cells are the main components that initiate, amplify and regulate acquired immunity. These cells maintain the immune memory and secrete cytokines to assist CD8+ T cells in killing tumor cells. Consistent with these roles, our study showed that the proportions of CD3+CD8+ T lymphocytes and CD3+CD4+ T cells were significantly increased in the peripheral blood of patient 3 after 6B11-OCIK immunotherapy. This result suggested that 6B11-OCIK immunotherapy promoted the proliferation and differentiation of T cells *in vivo*. We speculate that 6B11-OCIK might be able to improve immune function, rebuild anti-tumor specific immunity, and increase the T cell immune response *in vivo*.

Notably, this was only a preliminary and exploratory study, and the inclusion of only three patients is the main limitation of this study. The original plan for the trial was 10 samples, however, after the completion of 6B11 treatment in three patients, although one patient had SD, two patients had PD, and one of the patients showed rapid progression. This result indicated that 6B11-OCIK had a certain efficacy, but the efficacy of 6B11-OCIK alone was limited. And during follow-up (**Table 4**), we found that two patients were sensitive to chemotherapy after 6B11-OCIK treatment. We previously found that cell immunotherapy can improve the chemotherapy resistance of patients (unpublished data). In addition, previous studies showed that combining therapies can maximize the immune response as shown for various treatment regimens for ovarian cancer, such as immune check point inhibitors, anti-angiogenic VEGF antibody and poly (ADP-ribose) polymerase inhibitor (41–45). We thus speculate that cell therapy combined with chemotherapy might have a better outcome than cell therapy alone. Therefore, to maximize the benefit for patients, the follow-up clinical regimen will be changed to the combination of chemotherapy and cell therapy after communication with the Center for Drug Evaluation. Despite the small sample size, our data provide meaningful implications for 6B11-OCIK in terms of safety and effectiveness. Since there will no longer be the same therapy cases in the future, we decided to publish the three cases of data at first. Our current results provide important preliminary findings and will need to be further verified in future studies with a larger sample size.

In conclusion, this preliminary study indicated that 6B11-OCIK was safe and showed potential efficacy against platinum-resistant recurrent or refractory ovarian cancer. In addition to imaging and CA125 serum levels, the changes of CTC numbers correlated to the treatment response, and together with immune function estimation, it may provide an objective measure to evaluate the therapeutic effect of immunotherapy.

## Data Availability Statement

The original contributions presented in the study are included in the article/**Supplementary Material**. Further inquiries can be directed to the corresponding authors.

## Ethics Statement

This study was conducted in accordance with the Declaration of Helsinki, approved by the Institute Research Medical Ethics Committee of Peking University People’s Hospital (2017PHA107-01) and registered (NCT03542669) in June 2018 before the enrollment of the first participant in August 2018. All participants provided their written informed consent to participate in this study.

## Author Contributions

HYC undertook collection, analysis and interpretation of the data and wrote the manuscript drafts. RM helped designing the safety indicators and programs. HYC, SW, and XY collected the blood samples and performed CTC detection. YW critically revised the manuscript. TZ was responsible for the CT reports and response evaluation. YCL, YZhan, SL and YZhao prepared 6B11-OCIK and performed related detections *in vitro*. ZT, SD, YFW and HZ were responsible for recruiting volunteers and collecting clinical medical records. YL, HC and XC were responsible for the conception, design and performing of the whole study. All authors contributed to the article and approved the submitted version.

## Funding

This work was financially supported by the National Key Research and Development Program of China (No.2016YFA0201404), National Natural Science Foundation of China (No.81971360), the National Key Research and Development Program of China (2015BAI13B06), and the Beijing Science and Technology Planning Project of China (Z181100002218023).

## Conflict of Interest

Authors YW, YCL, YZhan, SL, and YZhan were employed by Beijing Weixiao Biotechnology Development Limited, Beijing, China.

The remaining authors declare that the research was conducted in the absence of any commercial or financial relationships that could be construed as a potential conflict of interest.

## Publisher’s Note

All claims expressed in this article are solely those of the authors and do not necessarily represent those of their affiliated organizations, or those of the publisher, the editors and the reviewers. Any product that may be evaluated in this article, or claim that may be made by its manufacturer, is not guaranteed or endorsed by the publisher.

## Supplementary Material

Supplementary Figure 1 Histopathologic images of ovarian tumor sections from the enrolled patients (Hematoxylin-eosin staining, 100x). **(A, B) Patient 1:** Specimens of ovarian tumor resection showed serous tumors with visible partial glandular duct and ingredient of papillary tumor, cell abnormal obviously, visible multi-core split phase (> 12/10 HPF), combined with immunohistochemical results, ovarian serous cancer tumor, high grade serous carcinomas. HE and immunohistochemical results: CK7 (+), PAX8 (+), p53 (-), p16 (+), WT2 (+), ER (15%+), PR (30%+), and Ki67 (70%+ in the cancer region). **(C, D) Patient 2:** The endometrium showed postmenopausal atrophy, but no tumor involvement was observed. The cervical mucosa showed chronic inflammation; cancer involvement was observed in the paracervical tissues, cancer invasion was observed in the mesentery, omentum and appendix, and no cancer involvement was observed in the adrenal tissues. Immunohistochemical staining results: CK7(+), CK20 (-), PAX8 (+), CA25 (+), p53 expression (-), WT1 (+), S-100 (+), SyN (-), CgA (-), CD56 (focal weak +), ER (-), PR (-), GATA-3 (-), HER-2 (-), Ki-67 (+ 30%), in line with high grade serous carcinoma. **(E, F) Patient 3:** Histological type: bilateral ovarian cancer, poorly differentiated; high grade serous carcinoma is considered. Tumor size: 10X8X6 cm on the left ovary; 5X4X2 cm on the right ovary. Fallopian tube:(left) no cancer, (right) with cancer. No cancer was observed in the omentum tissue. Lymph nodes: lymph nodes with cancer metastasis (4/5, 3/12); The mesentery, rectum, intestinal wall and sigmoid colon were found to have carcinoma, while the pelvic wall and peritoneum were suspected to have carcinoma. Immunohistochemical staining results: CK20 (-), CK7 (+), CA25 (+), WT1 (+), ER (+), PR (-), Napsina (-), P53 (+), Ki-67 (20%+), consistent with high grade serous carcinoma.

Supplementary Figure 2 Numbers of CTCs with chromosome 8 aneuploidy. The most prevalent aneuploidy for chromosome 8 of CTCs was pentaploid and above, followed by triploid, tetraploid, and haploid.

Supplementary Figure 3 Killing efficiency of 6B11-OCIK against the ovarian cancer cell line HOC1A. Five batches of 6B11-OCIK were obtained from each patient. The killing efficiency of each batch of 6B11-OCIK against the ovarian cancer cell line HOC1A increased with the increase of effect-target ratio. (The data in the fourth batch of patient 2 was lost due to equipment problems).

Supplementary Figure 4 Changes of peripheral blood lymphocyte phenotypes in patients during 6B11-OCIK treatment. During the treatment of 6B11-OCIK, the proportion of lymphocytes in peripheral blood of patient 1 **(A)** and patient 2 **(B)** barely changed, while the proportions of CD4+ and CD8+ T cells of patient 3 **(C);** increased.
