# Supplementary figures and images for: Preliminary Safety and Potential Effect of 6B11-OCIK Adoptive Cell Therapy Against Platinum-Resistant Recurrent or Refractory Ovarian Cancer
Source: Front Immunol. 2021 Aug 2;12:707468. doi: 10.3389/fimmu.2021.707468 (PMC8366315; doi:10.3389/fimmu.2021.707468)

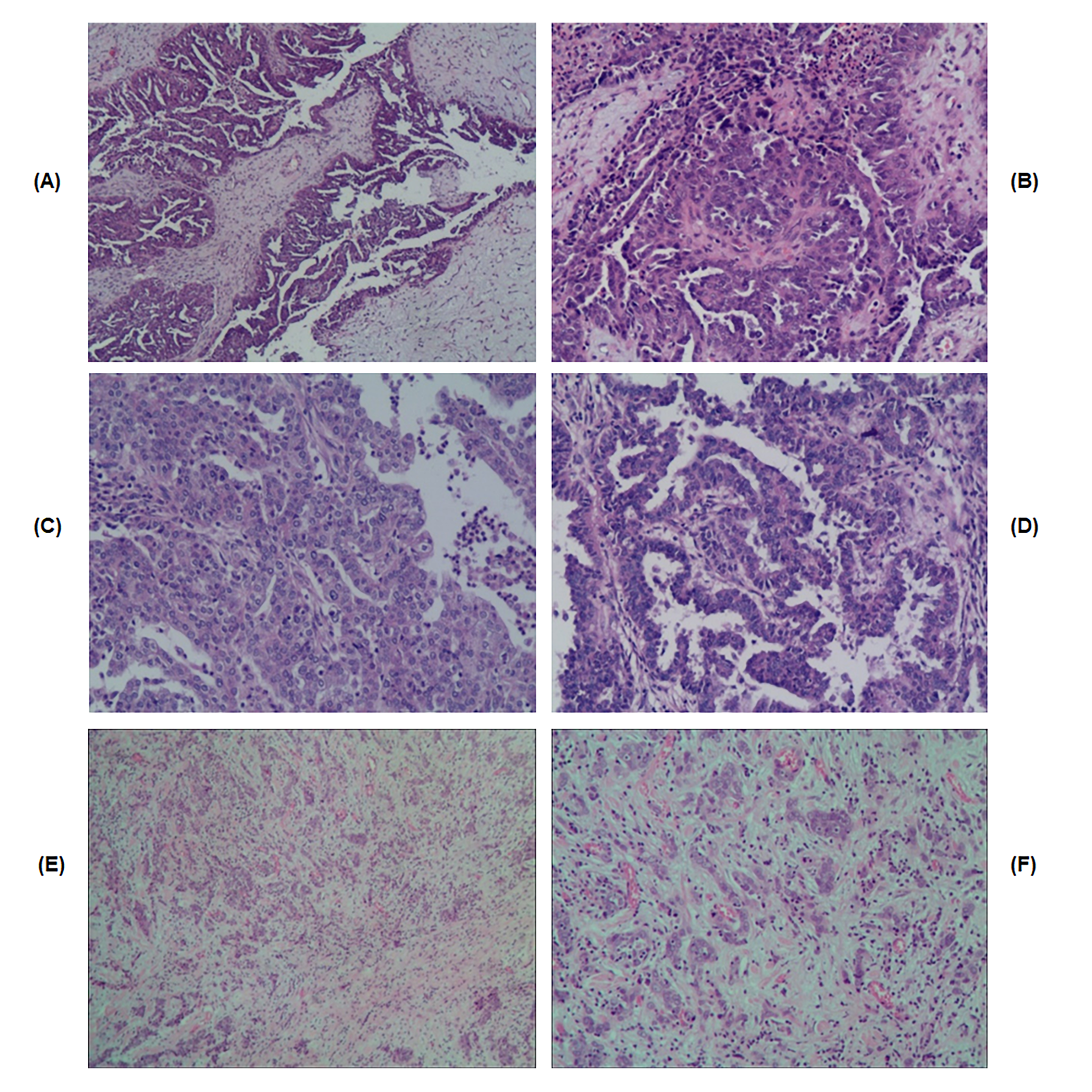

Supplement: Supplementary Figure 1 — Histopathologic images of ovarian tumor sections from the enrolled patients (Hematoxylin-eosin staining, 100x). (A, B) Patient 1: Specimens of ovarian tumor resection showed serous tumors with visible partial glandular duct and ingredient of papillary tumor, cell abnormal obviously, visible multi-core split phase (> 12/10 HPF), combined with immunohistochemical results, ovarian serous cancer tumor, high grade serous carcinomas. HE and immunohistochemical results: CK7 (+), PAX8 (+), p53 (-), p16 (+), WT2 (+), ER (15%+), PR (30%+), and Ki67 (70%+ in the cancer region). (C, D) Patient 2: The endometrium showed postmenopausal atrophy, but no tumor involvement was observed. The cervical mucosa showed chronic inflammation; cancer involvement was observed in the paracervical tissues, cancer invasion was observed in the mesentery, omentum and appendix, and no cancer involvement was observed in the adrenal tissues. Immunohistochemical staining results: CK7(+), CK20 (-), PAX8 (+), CA25 (+), p53 expression (-), WT1 (+), S-100 (+), SyN (-), CgA (-), CD56 (focal weak +), ER (-), PR (-), GATA-3 (-), HER-2 (-), Ki-67 (+ 30%), in line with high grade serous carcinoma. (E, F) Patient 3: Histological type: bilateral ovarian cancer, poorly differentiated; high grade serous carcinoma is considered. Tumor size: 10X8X6 cm on the left ovary; 5X4X2 cm on the right ovary. Fallopian tube:(left) no cancer, (right) with cancer. No cancer was observed in the omentum tissue. Lymph nodes: lymph nodes with cancer metastasis (4/5, 3/12); The mesentery, rectum, intestinal wall and sigmoid colon were found to have carcinoma, while the pelvic wall and peritoneum were suspected to have carcinoma. Immunohistochemical staining results: CK20 (-), CK7 (+), CA25 (+), WT1 (+), ER (+), PR (-), Napsina (-), P53 (+), Ki-67 (20%+), consistent with high grade serous carcinoma. [file Image_1.tif]

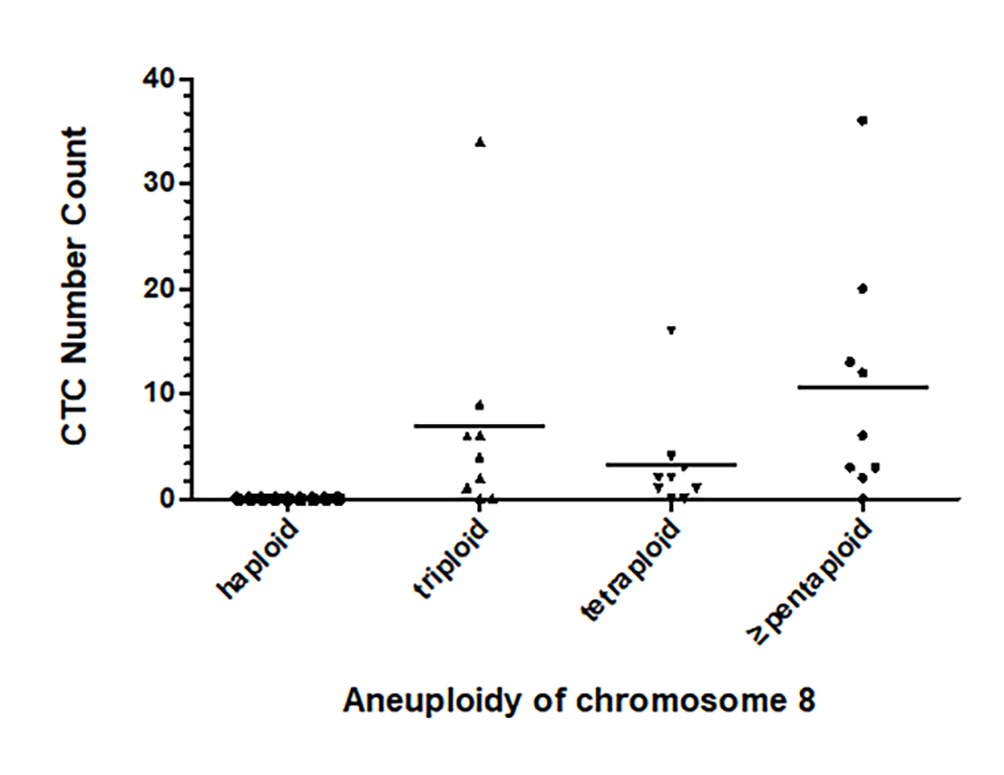

Supplement: Supplementary Figure 2 — Numbers of CTCs with chromosome 8 aneuploidy. The most prevalent aneuploidy for chromosome 8 of CTCs was pentaploid and above, followed by triploid, tetraploid, and haploid. [file Image_2.tif]

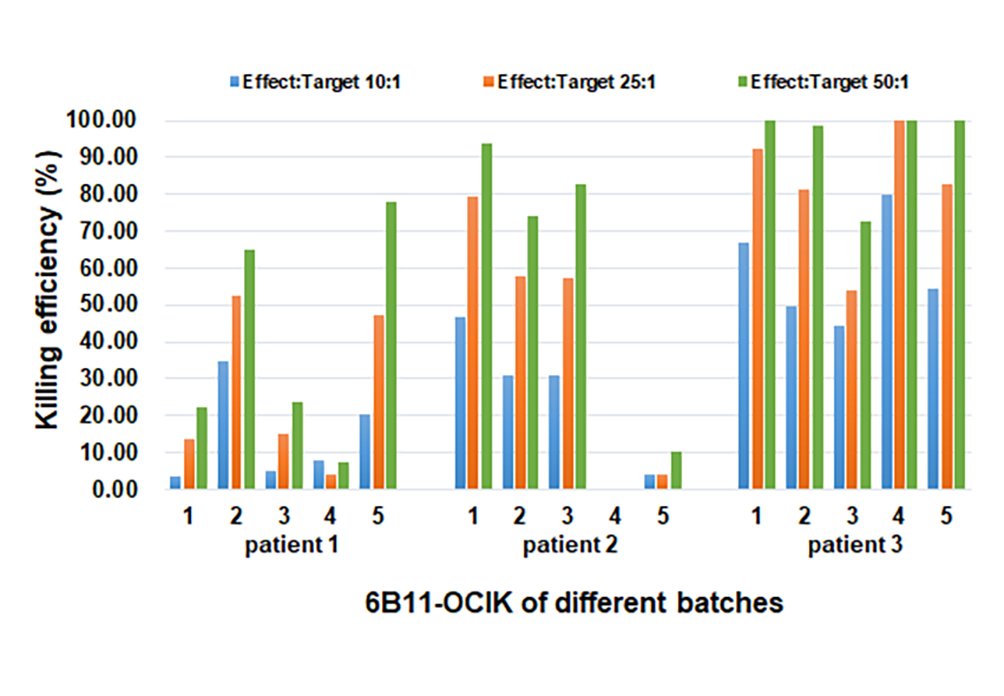

Supplement: Supplementary Figure 3 — Killing efficiency of 6B11-OCIK against the ovarian cancer cell line HOC1A. Five batches of 6B11-OCIK were obtained from each patient. The killing efficiency of each batch of 6B11-OCIK against the ovarian cancer cell line HOC1A increased with the increase of effect-target ratio. (The data in the fourth batch of patient 2 was lost due to equipment problems). [file Image_3.tif]

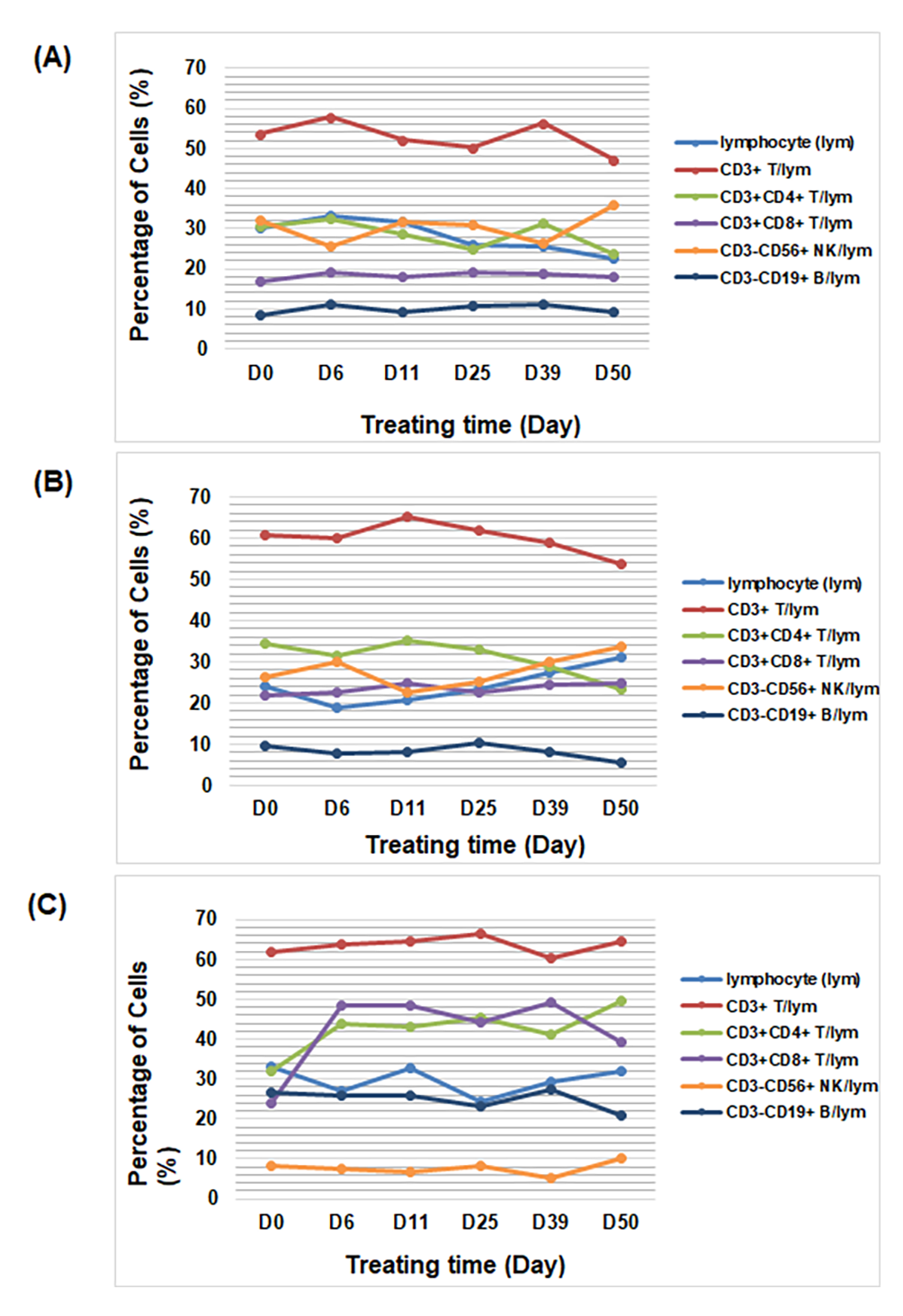

Supplement: Supplementary Figure 4 — Changes of peripheral blood lymphocyte phenotypes in patients during 6B11-OCIK treatment. During the treatment of 6B11-OCIK, the proportion of lymphocytes in peripheral blood of patient 1 (A) and patient 2 (B) barely changed, while the proportions of CD4+ and CD8+ T cells of patient 3 (C); increased. [file Image_4.tif]
