# Supplementary material for: Preliminary Safety and Potential Effect of 6B11-OCIK Adoptive Cell Therapy Against Platinum-Resistant Recurrent or Refractory Ovarian Cancer
Source: Front Immunol. 2021 Aug 2;12:707468. doi: 10.3389/fimmu.2021.707468 (PMC8366315; doi:10.3389/fimmu.2021.707468)
Supplement: Supplementary file 5 [file DataSheet_1.docx]

**Table S1．Variation of serum CA125 before and during 6B11-OCIK treatment**

| **Patients NO.** | **Tumor progression assessment (RECIST v 1.1)** | **Serum CA125 before 6B11-OCIK treatment(U/ml)** | **Variation of CA125 before 6B11-OCIK treatment(U/ml)** | **Serum CA125 during 6B11-OCIK treatment(U/ml)** | **Variation of CA125 during 6B11-OCIK treatment(U/ml)** |
| --- | --- | --- | --- | --- | --- |
| **1** | PD | 8/15/2016:322  8/21/2017:117  10/30/2017:566  3/8/2018:122 | instable | 8/16/2018:324.3 (D0)  9/17/2018:958.2 (D25)  10/12/2018:2347 (D50) | increase |
| **2** | PD | 9/25/2018:34.52  10/24/2018:91.61  11/17/2018:161.2 | increase | 11/28/2018:247.6 (D0)  12/29/2018:506.1 (D25)  1/17/2019:994 (D50) | increase |
| **3** | SD | 3/2/2018:127.6  3/29/2018:239.4  5/14/2018:308.5 | increase | 6/17/2019:380.4 (D0)  7/9/2019:292.1 (D25)  8/5/2019:283.5 (D50) | decrease |

*Normal serum CA125 level was ≤ 35U/ml.

PD：progressive disease

SD：stable disease

D0， D25，D50：About day0，day25 and day50 of 6B11-OCIK treatment.

D0，D25，D50：About day0，day25 and day50 of 6B11-OCIK treatment.

**Table S2. Variation of CTCs during 6B11-OCIK treatment**

| **Case No.** | **Treatment time** | **CTCs with different number of chromosome 8** | | | | | **Total number of single CTCs** | **Number of small CTCs (≤5 μm)** | **Circulating tumor emboli** | **Tumor marker positive CTC** | | |
| --- | --- | --- | --- | --- | --- | --- | --- | --- | --- | --- | --- | --- |
|  |  | **haploid** | **diploid** | **triploid** | **(≥2 cells)** | **≥** |  |  | **≥2cells)** | **HE4+** | **CA125+** | **HE4+&**  **CA125+** |
|  |  |  |  |  |  | **pentaploid** |  |  |  |  |  |  |
| **1** | **D0** | 0 | 0 | 0 | 0 | 0 | 0 | 0 | 0 | 0 | 0 | 0 |
|  | **D25** | 0 | 0 | 2 | 2 | 13 | 17 | 5 | 0 | 4 | 0 | 0 |
|  | **D50** | 0 | 0 | 6 | 1 | 3 | 10 | 7 | 0 | 0 | 0 | 0 |
| **2** | **D0** | 0 | 0 | 6 | 4 | 36 | 46 | 5 | 3 | 0 | 0 | 0 |
|  | **D25** | 0 | 0 | 1 | 1 | 2 | 4 | 2 | 0 | 1 | 0 | 0 |
|  | **D50** | 0 | 0 | 0 | 0 | 3 | 3 | 0 | 0 | 0 | 0 | 0 |
| **3** | **D0** | 0 | 0 | 34 | 16 | 20 | 70 | 50 | 0 | 0 | 1 | 0 |
|  | **D25** | 0 | 0 | 4 | 3 | 12 | 19 | 6 | 1 | 0 | 0 | 0 |
|  | **D50** | 0 | 0 | 9 | 2 | 6 | 17 | 8 | 0 | 0 | 0 | 0 |

| **Table S3. Cell proliferation detection of 6B11-OCIK** | | | | | | | | |
| --- | --- | --- | --- | --- | --- | --- | --- | --- |
| **Case No.** | **Product batch** | **Blood lymphocytes (×10^9^/L)** | **Volume of blood (ml)** | **PBMNC number (×10^7^)** | **Days**  **in culture** | **Amplification fold** | **Total cell number (×10^9^)** | **Cell viability (%)** |
|  |  |  |  |  |  |  |  |  |
| **1** | **1** | 1.85 | 108 | 8.37 | 13 | 35.84 | 1.50 | 92.90 |
|  | **2** |  |  |  | 19 | 57.83 | 2.42 | 97.60 |
|  | **3** |  |  |  | 26 | 79.57 | 3.33 | 96.50 |
|  | **4** | 1.48 | 135 | 5.4 | 19 | 23.33 | 1.26 | 98.20 |
|  | **5** | 1.39 | 144 | 11.2 | 13 | 38.04 | 4.26 | 92.80 |
|  | **SUM** | 1.57 | 129 | 8.32 | 18 | 46.92 | 2.55 | 95.60 |
|  | **SD** | 0.24 | 18.73 | 2.90 | 5.39 | 22.04 | 1.26 | 2.58 |
| **2** | **6** | 1.59 | 126 | 7.54 | 12 | 55.97 | 2.11 | 92.50 |
|  | **7** |  |  |  | 17 | 92.31 | 3.48 | 95.70 |
|  | **8** |  |  |  | 21 | 234.75 | 8.85 | 98.00 |
|  | **9** | 1.26 | 162 | 10.5 | 21 | 26.29 | 2.76 | 93.20 |
|  | **10** | 1.30 | 162 | 5.76 | 12 | 101.04 | 5.82 | 93.00 |
|  | **SUM** | 1.38 | 150 | 7.93 | 16.6 | 102.07 | 4.60 | 94.48 |
|  | **SD** | 0.18 | 20.78 | 2.39 | 4.51 | 79.95 | 2.76 | 2.33 |
| **3** | **11** | 2.47 | 81 | 6.08 | 12 | 59.21 | 1.80 | 97.80 |
|  | **12** |  |  |  | 16 | 118.09 | 3.59 | 98.10 |
|  | **13** |  |  |  | 21 | 309.87 | 9.42 | 98.70 |
|  | **14** | 2.22 | 90 | 5.43 | 12 | 35.36 | 1.92 | 97.00 |
|  | **15** | 2.78 | 72 | 8.66 | 11 | 67.21 | 5.82 | 98.00 |
|  | **SUM** | 2.49 | 81 | 6.72 | 14.4 | 117.95 | 4.51 | 97.92 |
|  | **SD** | 0.28 | 9.00 | 1.71 | 4.16 | 111.44 | 3.19 | 0.61 |

| **Table S4. Immunophenotypes of 6B11-OCIK and PBMNCs** | | | | | | | | | | | | | |
| --- | --- | --- | --- | --- | --- | --- | --- | --- | --- | --- | --- | --- | --- |
| **Case NO.** | **Cells** | **CD3+** | **CD3+**  **CD4+** | **CD3+**  **CD8+** | **CD3-CD56+** | **CD3+**  **CD56+** | **CD86+** | **CD80+** | **CD1α** | **CD83+** | **HLA-DR+** | **CD54+** | **CD40+** |
|  |  |  |  |  |  |  |  |  |  |  |  |  |  |
|  |  |  |  |  |  |  |  |  |  |  |  |  |  |
| 1 | **PBMNCs** | 55.07 | 29.05 | 18.85 | 33.04 | 4.90 | 8.05 | 1.31 | 0.10 | 1.31 | 59.74 | 84.02 | 5.13 |
|  | **6B11-OCIK** | 71.56 | 18.67 | 37.93 | 24.73 | 18.96 | 24.26 | 23.03 | 0.06 | 9.22 | 65.59 | 70.64 | 1.85 |
|  | **Increase Rate** | 16.49 | -10.38 | 19.08 | -8.31 | 14.06 | 16.21 | 21.72 | -0.05 | 7.91 | 5.85 | -13.38 | -3.28 |
| 2 | **PBMNCs** | 55.53 | 27.42 | 23.75 | 27.62 | 3.94 | 8.59 | 0.52 | 0.08 | 0.78 | 53.34 | 77.59 | 3.46 |
|  | **6B11-OCIK** | 87.85 | 36.34 | 45.69 | 2.06 | 12.59 | 23.25 | 7.21 | 0.07 | 2.19 | 79.22 | 77.52 | 1.70 |
|  | **Increase Rate** | 32.32 | 8.92 | 21.94 | -25.56 | 8.64 | 14.66 | 6.70 | 0.00 | 1.42 | 25.88 | -0.07 | -1.76 |
| 3 | **PBMNCs** | 64.12 | 33.25 | 27.43 | 6.61 | 3.80 | 4.33 | 0.62 | 0.03 | 0.19 | 53.12 | 73.69 | 25.93 |
|  | **6B11-OCIK** | 97.36 | 28.82 | 65.62 | 1.46 | 22.12 | 27.51 | 5.14 | 0.13 | 7.23 | 91.82 | 97.14 | 1.79 |
|  | **Increase Rate** | 33.24 | -4.43 | 38.19 | -5.16 | 18.32 | 23.18 | 4.52 | 0.10 | 7.04 | 38.70 | 23.45 | -24.14 |

Data are shown as %.

| **Table S5. Immunophenotypes of 6B11-OCIK and PBMNCs of patient 1** | | | | | | | | | | | | | | | |
| --- | --- | --- | --- | --- | --- | --- | --- | --- | --- | --- | --- | --- | --- | --- | --- |
| **Immunophenotypes** | **PBMNCs before culture** | | | | | | | **6B11-OCIK** | | | | | | | **Increased rate** |
|  | **1** | **2** | **3** | **4** | **5** | **Average** | **SD** | **1** | **2** | **3** | **4** | **5** | **Average** | **SD** |  |
| **CD3+** | 53.72 | 53.72 | 53.72 | 52.65 | 61.52 | 55.07 | 3.64 | 89.34 | 66.36 | 75.10 | 33.90 | 93.09 | 71.56 | 23.65 | 16.49 |
| **CD3+CD4+** | 28.23 | 28.23 | 28.23 | 26.62 | 33.96 | 29.05 | 2.83 | 34.73 | 11.09 | 2.90 | 0.92 | 43.71 | 18.67 | 19.40 | -10.38 |
| **CD3+CD8+** | 17.53 | 17.53 | 17.53 | 21.51 | 20.16 | 18.85 | 1.87 | 48.24 | 37.70 | 51.66 | 5.40 | 46.67 | 37.93 | 18.90 | 19.08 |
| **CD3-CD56+** | 33.72 | 33.72 | 33.72 | 37.23 | 26.82 | 33.04 | 3.80 | 7.66 | 24.98 | 21.46 | 63.30 | 6.25 | 24.73 | 23.08 | -8.31 |
| **CD3+CD56+** | 5.65 | 5.65 | 5.65 | 3.80 | 3.74 | 4.90 | 1.03 | 2.17 | 12.34 | 36.52 | 32.88 | 10.87 | 18.96 | 14.94 | 14.06 |
| **CD86+** | 8.42 | 8.42 | 8.42 | 5.10 | 9.90 | 8.05 | 1.77 | 23.18 | 33.21 | 30.90 | 2.83 | 31.19 | 24.26 | 12.58 | 16.21 |
| **CD80+** | 1.51 | 1.51 | 1.51 | 0.49 | 1.53 | 1.31 | 0.46 | 5.23 | 37.10 | 33.43 | 0.00 | 39.37 | 23.03 | 18.84 | 21.72 |
| **CD1α+** | 0.00 | 0.00 | 0.00 | 0.42 | 0.10 | 0.10 | 0.18 | 0.04 | 0.00 | 0.00 | 0.15 | 0.09 | 0.06 | 0.06 | -0.05 |
| **CD83+** | 1.55 | 1.55 | 1.55 | 1.14 | 0.77 | 1.31 | 0.35 | 0.00 | 23.01 | 10.30 | 0.35 | 12.43 | 9.22 | 9.56 | 7.91 |
| **HLA-DR+** | 61.29 | 61.29 | 61.29 | 58.75 | 56.06 | 59.74 | 2.33 | 91.28 | 23.18 | 97.68 | 23.80 | 92.00 | 65.59 | 38.51 | 5.85 |
| **CD54+** | 83.36 | 83.36 | 83.36 | 83.81 | 86.20 | 84.02 | 1.24 | 90.50 | 84.00 | 79.38 | 1.18 | 98.14 | 70.64 | 39.47 | -13.38 |
| **CD40+** | 5.19 | 5.19 | 5.19 | 3.88 | 6.18 | 5.13 | 0.82 | 0.31 | 0.51 | 0.09 | 8.02 | 0.30 | 1.85 | 3.45 | -3.28 |

Data are shown as %.

| **Table S6. Immunophenotypes of 6B11-OCIK and PBMNCs of patient 2** | | | | | | | | | | | | | | | |
| --- | --- | --- | --- | --- | --- | --- | --- | --- | --- | --- | --- | --- | --- | --- | --- |
| **Immunophenotypes** | **PBMNCs before culture** | | | | | | | **6B11-OCIK** | | | | | | | **Increased rate** |
|  | **1** | **2** | **3** | **4** | **5** | **Average** | **SD** | **1** | **2** | **3** | **4** | **5** | **Average** | **SD** |  |
| **CD3+** | 60.22 | 60.22 | 60.22 | 64.26 | 32.73 | 55.53 | 12.87 | 96.54 | 97.89 | 97.70 | 50.73 | 96.40 | 87.85 | 20.76 | 32.32 |
| **CD3+CD4+** | 29.90 | 29.90 | 29.90 | 34.83 | 12.57 | 27.42 | 8.57 | 60.81 | 36.88 | 27.09 | 13.55 | 43.37 | 36.34 | 17.70 | 8.92 |
| **CD3+CD8+** | 25.90 | 25.90 | 25.90 | 26.47 | 14.60 | 23.75 | 5.12 | 36.26 | 60.73 | 69.86 | 10.45 | 51.17 | 45.69 | 23.30 | 21.94 |
| **CD3-CD56+** | 35.54 | 35.54 | 35.54 | 28.40 | 3.09 | 27.62 | 14.06 | 1.85 | 1.60 | 2.01 | 1.95 | 2.91 | 2.06 | 0.50 | -25.56 |
| **CD3+CD56+** | 4.70 | 4.70 | 4.70 | 4.71 | 0.90 | 3.94 | 1.70 | 7.58 | 13.14 | 16.84 | 8.75 | 16.62 | 12.59 | 4.31 | 8.64 |
| **CD86+** | 5.41 | 5.41 | 5.41 | 14.24 | 12.50 | 8.59 | 4.40 | 27.28 | 29.64 | 22.21 | 0.00 | 37.14 | 23.25 | 14.07 | 14.66 |
| **CD80+** | 0.57 | 0.57 | 0.57 | 0.32 | 0.56 | 0.52 | 0.11 | 4.09 | 4.81 | 23.90 | 0.00 | 3.27 | 7.21 | 9.51 | 6.70 |
| **CD1α+** | 0.02 | 0.02 | 0.02 | 0.32 | 0.00 | 0.08 | 0.14 | 0.00 | 0.06 | 0.17 | 0.08 | 0.06 | 0.07 | 0.06 | 0.00 |
| **CD83+** | 1.01 | 1.01 | 1.01 | 0.03 | 0.82 | 0.78 | 0.43 | 2.06 | 0.21 | 3.63 | 1.79 | 3.28 | 2.19 | 1.36 | 1.42 |
| **HLA-DR+** | 51.64 | 51.64 | 51.64 | 64.34 | 47.44 | 53.34 | 6.41 | 85.41 | 92.59 | 95.47 | 26.97 | 95.64 | 79.22 | 29.50 | 25.88 |
| **CD54+** | 80.80 | 80.80 | 80.80 | 75.41 | 70.12 | 77.59 | 4.78 | 97.55 | 93.29 | 95.74 | 2.40 | 98.62 | 77.52 | 42.04 | -0.07 |
| **CD40+** | 3.00 | 3.00 | 3.00 | 5.65 | 2.64 | 3.46 | 1.24 | 1.19 | 0.33 | 0.40 | 5.42 | 1.15 | 1.70 | 2.12 | -1.76 |

Data are shown as %.

| **Table S7. Immunophenotypes of 6B11-OCIK and PBMNCs of patient 3** | | | | | | | | | | | | | | | |
| --- | --- | --- | --- | --- | --- | --- | --- | --- | --- | --- | --- | --- | --- | --- | --- |
| **Immunophenotypes** | **PBMNCs before culture** | | | | | | | **6B11-OCIK** | | | | | | | **Increased rate** |
|  | **1** | **2** | **3** | **4** | **5** | **Average** | **SD** | **1** | **2** | **3** | **4** | **5** | **Average** | **SD** |  |
| **CD3+** | 62.89 | 62.89 | 62.89 | 65.57 | 66.37 | 64.12 | 1.71 | 98.14 | 98.56 | 98.56 | 96.13 | 95.43 | 97.36 | 1.48 | 33.24 |
| **CD3+CD4+** | 32.31 | 32.31 | 32.31 | 34.27 | 35.07 | 33.25 | 1.32 | 32.09 | 19.24 | 14.27 | 39.99 | 38.52 | 28.82 | 11.54 | -4.43 |
| **CD3+CD8+** | 27.06 | 27.06 | 27.06 | 27.75 | 28.21 | 27.43 | 0.53 | 65.10 | 77.39 | 80.29 | 50.50 | 54.82 | 65.62 | 13.22 | 38.19 |
| **CD3-CD56+** | 7.65 | 7.65 | 7.65 | 5.49 | 4.62 | 6.61 | 1.45 | 1.15 | 1.02 | 1.21 | 2.64 | 1.26 | 1.46 | 0.67 | -5.16 |
| **CD3+CD56+** | 3.98 | 3.98 | 3.98 | 3.65 | 3.43 | 3.80 | 0.25 | 12.21 | 28.49 | 38.56 | 20.09 | 11.25 | 22.12 | 11.53 | 18.32 |
| **CD86+** | 6.71 | 6.71 | 6.71 | 0.84 | 0.68 | 4.33 | 3.26 | 26.87 | 25.13 | 13.46 | 40.10 | 31.99 | 27.51 | 9.77 | 23.18 |
| **CD80+** | 0.66 | 0.66 | 0.66 | 0.97 | 0.17 | 0.62 | 0.29 | 3.70 | 0.86 | 0.80 | 11.49 | 8.87 | 5.14 | 4.83 | 4.52 |
| **CD1α+** | 0.00 | 0.00 | 0.00 | 0.10 | 0.06 | 0.03 | 0.05 | 0.06 | 0.07 | 0.15 | 0.38 | 0.01 | 0.13 | 0.15 | 0.10 |
| **CD83+** | 0.21 | 0.21 | 0.21 | 0.22 | 0.10 | 0.19 | 0.05 | 11.01 | 1.55 | 2.48 | 17.18 | 3.94 | 7.23 | 6.69 | 7.04 |
| **HLA-DR+** | 56.34 | 56.34 | 56.34 | 47.84 | 48.74 | 53.12 | 4.42 | 92.58 | 90.53 | 83.69 | 95.30 | 97.01 | 91.82 | 5.18 | 38.70 |
| **CD54+** | 76.13 | 76.13 | 76.13 | 70.11 | 69.94 | 73.69 | 3.34 | 98.70 | 96.71 | 91.97 | 99.27 | 99.04 | 97.14 | 3.06 | 23.45 |
| **CD40+** | 24.87 | 24.87 | 24.87 | 27.20 | 27.86 | 25.93 | 1.48 | 3.72 | 0.42 | 0.04 | 2.52 | 2.26 | 1.79 | 1.53 | -24.14 |

Data are shown as %.

| **Table S8 Killing efficiency of 6B11-OCIK against the ovarian cancer cell line HOC1A** | | | | |
| --- | --- | --- | --- | --- |
| **Case No.** | **6B11-OCIK product batch** | **Killing efficiency (%)** | | |
|  |  | **Effect: Target 10:1** | **Effect: Target 25:1** | **Effect: Target 50:1** |
|  |  |  |  |  |
| **1** | **1** | 3.77 | 13.7 | 22.32 |
|  | **2** | 34.53 | 52.61 | 64.91 |
|  | **3** | 5.04 | 14.98 | 23.75 |
|  | **4** | 7.82 | 4.11 | 7.35 |
|  | **5** | 20.45 | 47.39 | 77.77 |
|  | **Average** | 14.32 | 26.56 | 39.22 |
|  | **SD** | 13.09 | 21.89 | 30.36 |
| **2** | **6** | 46.63 | 79.22 | 94.01 |
|  | **7** | 30.72 | 57.67 | 74.08 |
|  | **8** | 30.94 | 57.17 | 83 |
|  | **9** | – | – | – |
|  | **10** | 4.04 | 3.99 | 10.24 |
|  | **Average** | 28.08 | 49.51 | 65.33 |
|  | **SD** | 1.84 | 1.67 | 37.62 |
| **3** | **11** | 66.81 | 92.21 | 100 |
|  | **12** | 49.65 | 81.15 | 98.67 |
|  | **13** | 44.55 | 54.15 | 72.61 |
|  | **14** | 80.12 | 100 | 100 |
|  | **15** | 54.48 | 82.87 | 100 |
|  | **Average** | 59.12 | 82.08 | 94.26 |
|  | **SD** | 14.35 | 17.36 | 12.11 |

The data in the fourth batch of patient 2 (#9) were lost due to technical issues.
